# Dysfunction in the mitochondrial Fe-S assembly machinery leads to formation of the chemoresistant truncated VDAC1 isoform without HIF-1α activation

**DOI:** 10.1371/journal.pone.0194782

**Published:** 2018-03-29

**Authors:** Ioana Ferecatu, Frédéric Canal, Lucilla Fabbri, Nathalie M. Mazure, Cécile Bouton, Marie-Pierre Golinelli-Cohen

**Affiliations:** 1 Institut de Chimie des Substances Naturelles (ICSN), CNRS UPR 2301, Univ. Paris-Sud, Université Paris-Saclay, Gif-sur-Yvette, France; 2 Institute for Research on Cancer and Aging of Nice, CNRS-UMR 7284-Inserm U1081, University of Nice Sophia-Antipolis, Centre Antoine Lacassagne, Nice, France; Institut de Pharmacologie Moleculaire et Cellulaire, FRANCE

## Abstract

Biogenesis of iron-sulfur clusters (ISC) is essential to almost all forms of life and involves complex protein machineries. This process is initiated within the mitochondrial matrix by the ISC assembly machinery. Cohort and case report studies have linked mutations in ISC assembly machinery to severe mitochondrial diseases. The voltage-dependent anion channel (VDAC) located within the mitochondrial outer membrane regulates both cell metabolism and apoptosis. Recently, the C-terminal truncation of the VDAC1 isoform, termed VDAC1-ΔC, has been observed in chemoresistant late-stage tumor cells grown under hypoxic conditions with activation of the hypoxia-response nuclear factor HIF-1α. These cells harbored atypical enlarged mitochondria. Here, we show for the first time that depletion of several proteins of the mitochondrial ISC machinery in normoxia leads to a similar enlarged mitochondria phenotype associated with accumulation of VDAC1-ΔC. This truncated form of VDAC1 accumulates in the absence of HIF-1α and HIF-2α activations and confers cell resistance to drug-induced apoptosis. Furthermore, we show that when hypoxia and siRNA knock-down of the ISC machinery core components are coupled, the cell phenotype is further accentuated, with greater accumulation of VDAC1-ΔC. Interestingly, we show that hypoxia promotes the downregulation of several proteins (ISCU, NFS1, FXN) involved in the early steps of mitochondrial Fe-S cluster biogenesis. Finally, we have identified the mitochondria-associated membrane (MAM) localized Fe-S protein CISD2 as a link between ISC machinery downregulation and accumulation of anti-apoptotic VDAC1-ΔC. Our results are the first to associate dysfunction in Fe-S cluster biogenesis with cleavage of VDAC1, a form which has previously been shown to promote tumor resistance to chemotherapy, and raise new perspectives for targets in cancer therapy.

## Introduction

In mammals, iron-sulfur (Fe-S) clusters are essential cofactors for numerous proteins involved in critical cellular functions, including electron transfer for oxidative phosphorylation, ribosome biogenesis, and DNA synthesis and repair [1]. Cluster maturation of all Fe-S proteins, independently of their subcellular localization, starts in the mitochondria and involves a complex iron-sulfur cluster (ISC) assembly machinery. First, the iron is imported into mitochondria by the carrier proteins mitoferrin 1 and 2 (MFRN-1 and -2), and inorganic sulfide is supplied from L-cysteine by cysteine-desulfurase NFS1 complexed to ISD11 and acyl carrier protein (ACP) [2]. Then, a [2Fe-2S] cluster is assembled on the scaffold protein ISCU with the help of frataxin (FXN) and of the ferredoxin/ferredoxin reductase reducing system. This transiently bound [2Fe-2S] can be transferred to mitochondrial [2Fe-2S]-assembling recipient apo-proteins with the participation of the chaperone and co-chaperone HSPA9/HSC20 [3,4] and glutaredoxin 5 [5]. Alternatively, it can either serve for the synthesis of [4Fe-4S] clusters and their insertion into mitochondrial [4Fe-4S]-assembling recipients (*e*.*g*. the Fe-S-containing respiratory complex subunits, the aconitase of the Krebs cycle) or it can be exported out of mitochondria *via* an ill-defined ISC export machinery for the maturation of extra-mitochondrial Fe-S proteins by the cytosolic assembly machinery (CIA) [1]. An efficient mitochondrial Fe-S cluster biogenesis pathway is required to maintain mitochondrial activity. Then, mutations in its components cause severe diseases currently characterized by mitochondrial dysfunction [6]. Interestingly, Fe-S proteins including NEET proteins have recently been linked to response mechanisms to cell damage, including apoptosis and autophagy regulation [7–9].

The voltage-dependent anion-selective channel (VDAC), also known as mitochondrial porin, is a channel-forming protein located in the outer mitochondrial membrane (OMM). VDAC is at a critical position between the mitochondrial inter-membrane space (IMS) and the cytosol, and enables metabolite exchange across the OMM, such as small hydrophilic anions, Ca^2+^ and adenine nucleotides necessary for the appropriate function of the mitochondrial respiratory chain [10,11]. Beside its role in basal cell functioning, VDAC is also a key player in mitochondria-mediated apoptosis [12] and a converging target of both pro- and anti-apoptotic Bcl-2 family proteins. Three isoforms of VDAC (VDAC1, VDAC2 and VDAC3), encoded by three different genes, have been identified in mammals [13]. VDAC1 and VDAC3 are pro-apoptotic and by protein oligomerization can form large pores that favor OMM permeabilization and release of apoptogenic molecules residing in the IMS, such as cytochrome c [14]. In contrast, the VDAC2 isoform is rather anti-apoptotic by interacting with BAK, a pro-apoptotic member of the Bcl2 family and regulating BAK-mediated cell death [15].

Limiting oxygen conditions (between 3 and 0.1%), which are called hypoxia, is a characteristic feature of many solid tumor microenvironments and often triggers molecular pathways that are responsible for tumor resistance to chemotherapy-induced apoptosis [16]. Recently, a C-terminal end truncated form of VDAC1, called VDAC1-ΔC, with an apparent molecular weight of 25 kDa on SDS-PAGE under reducing conditions, was detected in tumor cells grown under oxygen-deprived conditions [17]. This truncation involves a lysosomal endopeptidase localized at the membrane contact sites between mitochondria and lysosomes [18]. Accumulation of this hypoxic VDAC1-ΔC was associated with resistance of several cancer cell lines to apoptosis induced by chemotherapeutic agents due to interaction of VDAC1-ΔC with Bcl-X_L_ and hexokinase II, two anti-apoptotic proteins. Although the mitochondrial inner transmembrane potential (ΔΨ_m_) was unchanged, the protective phenotype was mainly attributed to major modifications in mitochondrial morphology, including cristae remodeling and formation of enlarged mitochondria due to imbalance in mitochondrial membrane fusion/fission dynamic processes [19]. Up to now, the formation of the truncated VDAC1-ΔC was demonstrated only under hypoxic conditions involving the stabilization and activation of the nuclear transcription factor hypoxia-inducible factor-1α (HIF-1α), which controls cell adaptation to low-oxygen conditions. Here, for the first time, we show that deficiency in mitochondrial ISC assembly machinery leads to the accumulation of VDAC1-ΔC concomitant with the appearance of an enlarged mitochondrial phenotype even when cells are grown under normoxic conditions (21% O_2_). This phenotype was observed without HIF-1α stabilization and activation and conferred cell resistance to apoptosis induction, as previously observed with tumor cells grown in hypoxia [20]. Combining depletion of ISC assembly machinery or iron chelator treatment with hypoxic conditions (1% O_2_) led to even greater accumulation of the VDAC1-ΔC form. We also underlined that hypoxia down-regulates several proteins of the mitochondrial ISC assembly machinery. Finally, we focused on CISD2, a Fe-S protein with a strategic localization at the contact sites between the OMM and the endoplasmic reticulum (ER), which indirectly interacts with VDAC1 [21,22]. We found out that specific depletion of this protein also induced the accumulation of VDAC1-ΔC, implying that CISD2 plays a pivotal role in the fate of VDAC1, a potential mitochondrial marker for cancer severity and prognosis.

## Materials and methods

### Cell culture and treatment

Human epithelial carcinoma cells (HeLa), liver hepatocellular carcinoma cells (HepG2) and human breast adenocarcinoma (MDA-MB-231) cells were cultured in Dulbecco’s modified Eagle medium (DMEM, Sigma-Aldrich) containing 4.5 g/L glucose, 1 mM stable L-glutamine and supplemented with 1% penicillin-streptomycin and 10% fetal bovine serum (Lonza) under 5% CO_2_ and humidified atmosphere. For hypoxic conditions, cells were grown at 37°C in a 1% O_2_, 94% nitrogen and 5% CO_2_ atmosphere for 3 and/or 6 days, as specified. Ferric ammonium citrate (FAC, 100 μM), desferrioxamine (DFO, 100 μM), CoCl_2_ (200 μM, the treatment is renewed after 24 h) and staurosporine (STS, 1 μM) were from Sigma-Aldrich. Salicylaldehyde isonicotinoyl hydrazone (SIH, 10–100 μM) was a kind gift from P. Ponka (McGill University, Montreal). NO donor diethylenetriamine NONOate (DETA-NO, 50–250 μM) was from Cayman Chemical.

### Epifluorescence and confocal microscopy

Cells grown on glass coverslips were incubated with 25 nM mitochondrial selective probe MitoTracker^®^ Red CMXRos (Invitrogen) for 30 min and fixed in 10% formalin solution (Sigma-Aldrich) for 10 min at 37°C. Nuclei were stained with Hoechst 33342 (1 μg/mL) for 5 min. Coverslips were mounted in a Citifluor AF2 solution (Biovalley) and cells were examined by epifluorescence under a Nikon confocal microscope. Image treatment was done using Nikon EZ-C1 software.

### Preparation of cell extracts and western blot

Total protein extracts from human cell lines were obtained by harvesting cells in Laemmli buffer (0.06 M Tris-HCl, pH 6.8, 10% glycerol, 2% SDS, protease inhibitors (Calbiochem)). Protein concentrations were determined using the BCA method. Equal amounts of proteins (50 μg) were separated on SDS-PAGE and transferred on 0.45 μm PVDF membranes. The primary antibodies used were: anti-β-actin (Sigma-Aldrich #A5441), -CAIX (carbonic anhydrase 9, NovusBio #NB100-417SS), -CIAPIN1 (Sigma-Aldrich #HPA042182), -cleaved caspase-3 (Cell Signaling #9661), -caspase-9 (5B4) (Abcam #Ab28131), -CISD2 (Proteintech #13318-1-AP), -FXN (frataxin, a gift from Dr. H. Puccio IGBMC, Illkirch, France), -HIF-1α (NovusBio #NB100-449), -HSC20 (Sigma-Aldrich #HPA018447), -ISCU (Proteintech #14812-1-AP), -mitoNEET (designed by Eurogentec), -NARFL (Sigma-Aldrich #HPA040851), -NFS1 (Proteintech #15370-1-AP), -NUBP1 (Sigma-Aldrich #HPA041656), -PARP1 (Calbiochem #AM30), -VDAC1 Nter (Interchim #AP17825PU-N), -VDACs poly (recognizing VDAC1, 2 and 3 isoforms, a gift from Dr. C. Brenner, INSERM U769, University of Paris-Sud, France), -HIF-2α (Novus #NB100-122) and–VINCULIN (Sigma-Aldrich #V9131). Secondary antibodies were anti-mouse, anti-rabbit and anti-chicken fluorescent IRDye 800CW (LI-COR) and membranes were scanned with an Odyssey^®^ Imaging System (LI-COR). Quantitation was performed using Li-Cor Odyssey software. In some blot images, unnecessary lanes were cut off and clearly demarcated using black lines in the corresponding figures.

### Quantitative real-time PCR analysis

Total RNA from cells was extracted using Direct-zol^™^ RNA system according to the manufacturer’s protocol (Zymo Research), and the reverse transcription of 1 μg of total RNA was performed using the High Capacity cDNA Archive Kit (Applied Biosystems). Quantitative real-time PCR was performed using the FastStart DNA master plus SYBR green I kit and the Roche Lightcycler system (Roche Applied Sciences). Primer sequences used were: Hu-*fxn* (forward: 5’-AGAGGAAACGCTGGAACTCTT-3’; reverse: 5’-ACGCTTAGGTCCACTGGATG-3’), Hu-*iscu2* (forward: 5’-CCCGACTCTATCACAAGAAGGTTG-3’; reverse: 5’-CATGCTGGAGCCCCCAC-3’), Hu-*vegf A* (forward: 5’- AAGCCCATGAAGTGGTGAAG-3’; reverse: 5’-TATGTGCTGGCTTTGGTGAG-3’), Hu-*ca9* (forward: 5’- AGGATCTACCTACTGTTGAG-3’; reverse: 5’-TGGTCATCCCCTTCTTTG-3’), Hu-*oct4* (forward: 5’–GCTTTGCATATCTCCTGAAG-3’; reverse: 5’- GATCACCCTGGGATATACAC-3’; Hu-*glut-1* (forward: 5’–TCACACTTGGGAATCAGCCCC-3’; reverse: 5’- TTCACTGTCGTGTCGCTCTTTG-3’).

Sequence-specific primers were designed to span intron-exon boundaries to generate amplicons of approximately 100 bp. Values were normalized to 18S rRNA and GAPDH mRNA.

### siRNA transfections

HeLa and MDA-MB321 cells were seeded at 3.5 × 10^5^ cells/cm^2^, incubated overnight and transfected with siRNA duplexes with INTERFERin^™^ (Polyplus Transfection) according to the manufacturer’s recommendations (Life Technologies^®^). For incubations with siRNA longer than three days, cells were transfected every three days. The siRNA duplexes were from Life Technologies^®^: *iscu* (s23909), *nfs1* (s17265), *mfrn*-2 (s37872), *hsc20* (s45405), *cisd2* (s54620), negative control (#4390843) and used at a final concentration of 10 nM.

### Cell fractionation

Mitochondria were prepared using a conventional differential centrifugation procedure. Briefly, HepG2 cells were seeded in 140 mm dishes and treated with DMSO (control) or with 50 μM SIH for 16 h. Cells were collected and washed with PBS buffer and allowed to swell for 15 min in an ice-cold hypotonic buffer (250 mM sucrose, 0.1 mM EDTA, 1 mM EGTA, 10 mM Hepes-KOH, pH 7.4, protease inhibitor cocktail (Sigma-Aldrich)). Cell disruption was performed by passing cells in a Dounce homogenizer. The homogenate was spun at 700 ×*g* for 10 min at 4°C to remove nuclei and debris, and the supernatant was spun at 10,000 ×*g* for 20 min at 4°C to separate the mitochondrial (pellet) from the cytosolic fraction (supernatant). The mitochondrial pellets were washed with isotonic buffer for mitochondria (250 mM sucrose, 5 mM succinate, 5 mM KH_2_PO_4_, 10 mM Hepes-KOH pH 7.4, protease inhibitor cocktail), spun again at 10,000 ×*g* for 20 min at 4°C and extracted in Laemmli buffer to a final concentration of 2–3 μg/μL. VDAC and VDAC1-ΔC protein levels and controls for each fraction (mitoNEET for mitochondrial and NUBP1 for cytosolic fractions) were assayed by immunoblotting.

### Mitochondrial membrane potential

Cells were analyzed for hallmarks of mitochondrial depolarization by using the membrane-permeable JC-1 (5,5’,6,6’-tetrachloro-1,1’,3,3’ tetraethylbenzimidazolylcarbocyanine iodide) dye according to the manufacturer’s protocol (Biotium). Flow cytometry analysis was performed on an FC500 Beckman Coulter instrument.

*Statistical analysis*- All results are presented as the mean +/- standard deviation of at least three independent experiments. Data were analyzed using one-way ANOVA analysis of variance with SigmaPlot. The Dunnett’s test was performed for all multiple comparisons versus control group. The Student-Newman-Keuls test was used for all pairwise comparisons of mean responses among the different treatment groups. Differences between groups were considered significant if the p value was less than 0.05, and mentioned in the figure legends.

## Results

### Knock-down of proteins of the mitochondrial ISC machinery causes hypoxic-like macro-mitochondria and accumulation of a truncated VDAC1 isoform, VDAC_25K_

**Fig 1:**
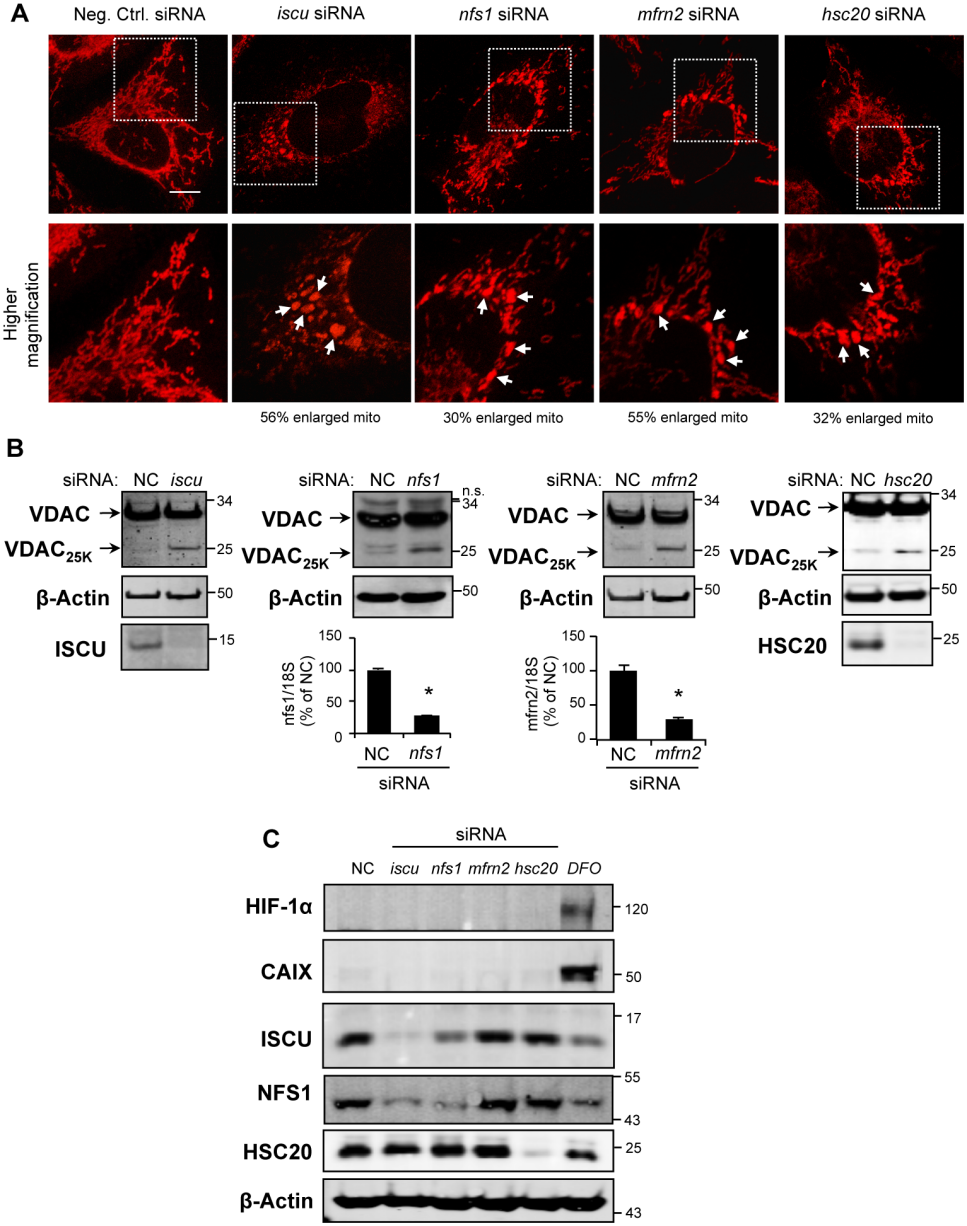
Depletion of proteins of the mitochondrial ISC assembly machinery leads to the formation of enlarged mitochondria without HIF-1α activation. HeLa cells were transfected with either negative control (NC), or *iscu-*, *nfs1-*, *mfrn2-* or *hsc20*-siRNA for 6 days. (A) Confocal microscopy after CMXRos staining to visualize mitochondria *(upper panels)*. Scale bar: 10 μm. *(lower panels)* Higher magnification of the part of the upper panel image delineated by a white square. (B) 6 days after transfection, total protein extracts were analyzed by immunoblotting using VDACs poly antibody detecting all isoforms of VDAC, and antibodies against ISCU or HSC20. β-Actin was used as loading control. The mRNA levels of *nfs1* and *mfrn2* were determined by RT-qPCR 24 h after transfection. Data are normalized to *18S* ribosomal rRNA levels and represented as a percentage of NC ± S.D. *, p < 0.001 (n = 3). (C) Cells were transfected with the specified siRNA for 6 days or treated with desferrioxamine (DFO) for 16 h. Immunoblotting was carried out to determine HIF-1α, CAIX, ISCU, NFS1 and HSC20 protein levels in total extracts. β-Actin is shown as loading control.

Several Fe-S proteins perform critical functions in the mitochondrion, such as mitochondrial respiration (Fe-S subunits of complexes I, II and III) and the Krebs cycle (mitochondrial aconitase). Therefore, to examine the effects of ISC deficiency on mitochondrial network, we used different siRNAs to selectively deplete the iron carrier MFRN2 or proteins of the mitochondrial ISC machinery (NFS1, ISCU and HSC20) in HeLa cells grown in normoxia (21% of O_2_). We knew from previous studies that most of the proteins involved in the maturation of Fe-S cluster are quite stable and up to 6 days of mRNA depletion are necessary to see highest level of depletion of the corresponding protein. Then, six days after transfection, living cells were stained with CMXRos probe to visualize the mitochondrial network (Fig 1A). In comparison with control cells (cells transfected with a negative control siRNA, Neg. Ctrl. siRNA), knockdown of MFRN2 and of ISC proteins (ISCU, NFS1 and HSC20) led to aberrant mitochondrial distribution, loss of the network and formation of mostly perinuclear aggregates of enlarged mitochondria (56%, 30%, 55%, and 32% of the cells have enlarged mitochondria when *iscu*, *nfs1*, *mfrn2*, and *hsc20* were respectively knocked-down). However, CMXRos probe staining (Fig 1A) still suggested maintenance of a mitochondrial transmembrane potential (ΔΨm). Moreover, flow cytometry analysis after JC-1 staining confirmed that, even in the more drastic conditions (6-day treatment with *iscu* siRNA), ΔΨm was not significantly affected (S1 Fig). Efficiency of the silencing was confirmed either by immunoblotting (ISCU and HSC20) or by RT-qPCR (*nfs1* and *mfrn2*) 24H after transfection due to the fact that mRNA depletion using siRNA is a fast process (Fig 1B). Then, we checked with another human cancer cell line, the breast cancer MDA-MB-231 cells, that similar observations could be made. We depleted *iscu* for 6 days using siRNA and looked at the mitochondrial network using CMXRos probe staining. As for HeLa cells, depletion of ISCU protein led to the formation of giant mitochondria with a disturbed mitochondrial network (S2 Fig).

As similar enlarged mitochondria with cristae reorganization have been observed in tumor cells grown in hypoxia [17], HeLa cells were submitted to 1% O_2_ for 5 days and the mitochondrial network was compared with that obtained after ISC assembly depletion in normoxia (S3 Fig and Fig 1A). Interestingly, enlarged mitochondria were observed similar to those obtained by silencing *iscu*, *nfs1*, *mfrn2* or *hsc20*.

In tumor cells grown in hypoxia, this mitochondrial phenotype was previously linked to a C-terminal truncation of VDAC1 (VDAC1-ΔC form) with an apparent migration of 25 kDa on reducing SDS-PAGE gel [17,18,23]. Using a polyclonal anti-VDAC antibody (VDACs poly), which detects all three isoforms of VDAC, we observed that depletion of MFRN2 and proteins of the ISC assembly machinery (ISCU, NFS1, HSC20) induced the formation of a truncated form of VDAC (VDAD_25K_) in normoxia in HeLa cells (Fig 1B) with a migration on SDS-PAGE similar to that of VDAC1-ΔC observed previously in cells grown in hypoxia. Kinetics studies of the siRNA treatments were performed and showed that VDAD_25K_ accumulated over time when depletion of MFRN2, ISCU, NFS1 and HSC20 was maintained (S4 Fig). A 6-day *iscu* or *mfrn2* siRNA treatment induced 6.0+/- 2.3% and 3.9+/-0.9%, of VDAC_25K_ accumulation, respectively, a cleavage level equivalent to what we obtained with HeLa cells under hypoxic conditions (5.2 +/- 1.4%). This truncated form is barely detected in cells transfected with control siRNAs (Fig 1B and S4 Fig).

In order to discriminate the intrinsic role of Fe-S deficiency towards hypoxia in the formation of VDAD_25K_, we then studied whether depletion of MFRN2 and of mitochondrial ISC assembly proteins in HeLa cells in normoxia induces the stabilization of hypoxia central transcription factor HIF-1α (Fig 1C) or the expression of one of its main transcriptional targets, carbonic anhydrase IX, at both mRNA (*ca9*, S5 Fig) and protein levels (CAIX, Fig 1C), as compared with cells treated overnight with the hypoxia-mimetic agent desferrioxamine (DFO) [24]. As shown in Fig 1C, in HeLa cells grown in normoxia, depletion of the iron importer MFRN2, of core ISC assembly proteins (ISCU and NFS1) or of the chaperone HSC20 induced formation of enlarged mitochondria associated with accumulation of VDAC_25K_ without stabilization of HIF-1α. Then, we also verified that HIF-2α was not stabilized at the protein level (S6 Fig, panel A) and that two of its main target genes (*glut1* and *oct4*) were not induced (S6 Fig, panel B).

### Fe-S cluster assembly deficiency leads to truncation of VDAC1

**Fig 2:**
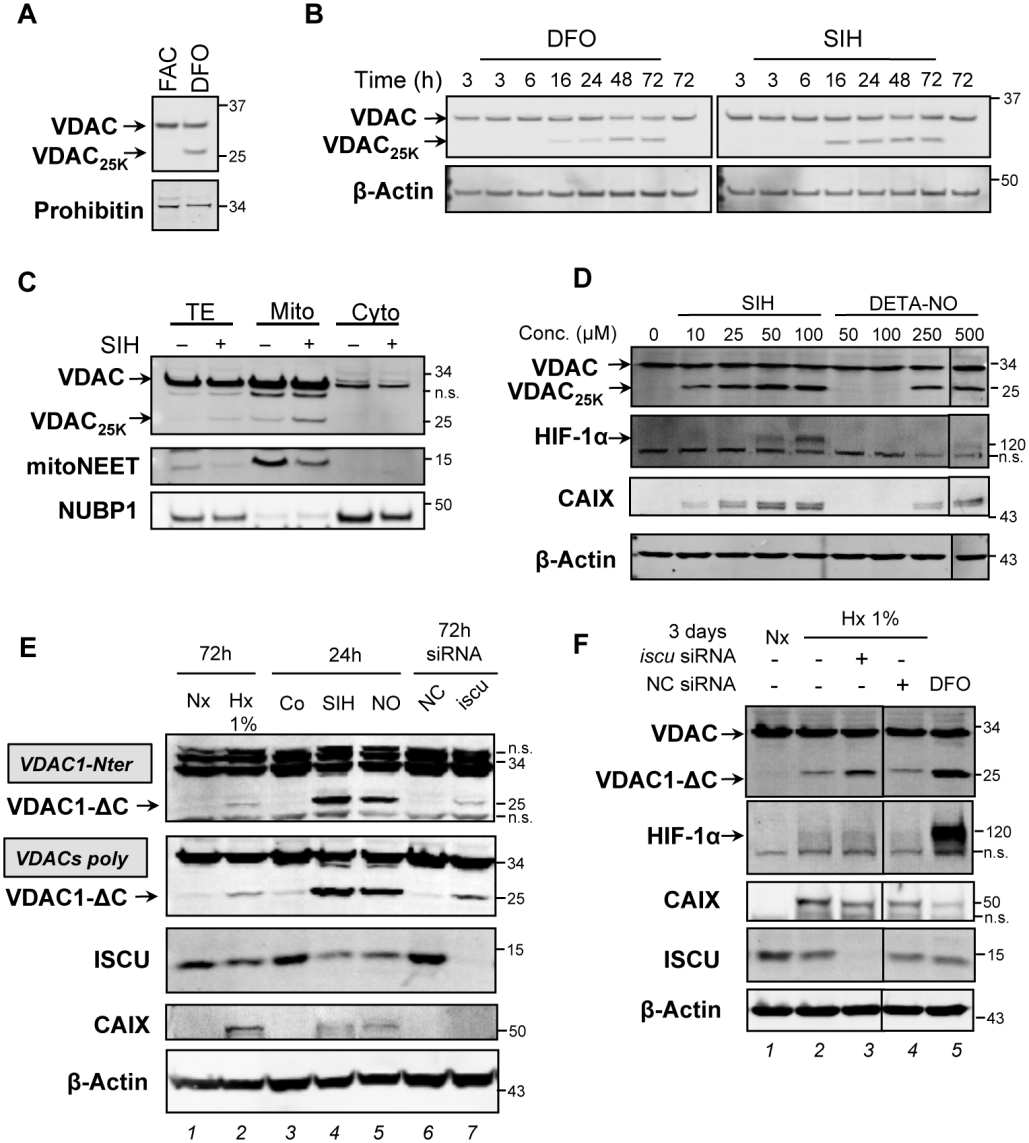
Iron depletion and nitric oxide stress induce the accumulation of the truncated VDAC1 form. (A) Total protein extracts of HeLa cells treated for 16 h with FAC or DFO were analyzed by immunoblotting using VDACs poly antibody. Prohibitin was used as loading control. (B) Total protein extracts of HeLa cells treated with DMSO (control), DFO or SIH for the indicated times were analyzed by immunoblotting using VDACs poly antibody. β-Actin antibody was used as loading control. (C) HepG2 cells were treated for 24 h with DMSO (control) or SIH. Immunoblotting was carried out using VDACs poly antibody on total protein extracts (TE), and on mitochondrial (Mito) and cytosolic (Cyto) fractions. mitoNEET and NUBP1 were used as mitochondrial and cytosolic markers, respectively. (D). HeLa cells were treated for 16 h with DMSO (control), SIH or DETA-NO (NO). Total protein extracts were analyzed by immunoblotting using VDACs poly antibody and anti-HIF-1α and -CAIX antibodies. β-Actin was used as loading control. (E) HeLa cells were grown in normoxia (Nx) or 1% O_2_ hypoxia (Hx) conditions for 3 days, or grown in normoxia conditions and treated for 24 h with DMSO (Control, Co), SIH or DETA-NO (NO), or grown in normoxia (21% O_2_) conditions and transfected with either negative control (NC) or *iscu*-siRNA for 3 days. Total protein extracts were analyzed by immunoblotting using antibodies against the N-terminus of VDAC1 isoform (VDAC1-Nter), the three VDAC isoforms (VDACs poly), ISCU and CAIX. (F) HeLa cells were grown in hypoxia (Hx, 1% O_2_) conditions and transfected with *iscu-* or NC-siRNA for 3 days, or grown in normoxia (Nx, 21% O_2_) and treated or not with DFO for 16 h. Total proteins were analyzed by western blot using VDACs poly antibody and anti-HIF-1α, -CAIX, -ISCU antibodies. β-Actin was used as loading control.

Cellular iron availability is central for Fe-S cluster biogenesis as iron deprivation leads to Fe-S cluster deficiency. Interestingly, it is well known that intracellular iron deficit can mimic hypoxia through inhibition of iron-dependent prolyl-4-hydroxylases [25–29]. However, the impact of cellular iron availability on the fate of VDAC has never been tested before, so we examined its impact on the formation of VDAC_25K_ in living cells. First, cells were exposed to ferric ammonium citrate (FAC) to induce intracellular iron overload or to DFO to deplete cellular iron. Formation of VDAC_25K_ is largely induced by overnight DFO treatment, while the truncated form is not observed after similar FAC treatment (Fig 2A). Furthermore, we decided to compare the kinetics of VDAC_25K_ formation induced by two iron chelators, DFO and salicylaldehyde isonicotinoyl hydrazone (SIH), which is a more cell permeable chelator. After 16 h of treatment, VDAC_25K_ appeared with both treatments with a stronger effect for SIH (Fig 2B). We noticed that the formation of VDAC_25K_ in iron-deficient cells is faster than observed previously in hypoxia [17]. In both cases, the protein level of VDAC_25K_ remained stable, up to at least 3 days (Fig 2B). We then examined the sub-cellular location of VDAC_25K_ in iron-deficient cells by performing cell fractionation after overnight treatment with SIH. The purity of mitochondrial-enriched and cytosolic fractions was assessed using the outer mitochondrial membrane protein mitoNEET and the cytosolic NUBP1 (Fig 2C). Clearly, VDAC_25K_ is found in the mitochondrial-enriched fraction as VDAC1 and the hypoxia-induced VDAC1-ΔC [17]. Moreover, we also used a nitric oxide-releasing compound, which is well known to trigger Fe-S cluster disassembly [30–32], to assess the fate of VDAC. We showed that VDAC_25K_ also accumulated in cells treated with DETA-NO, an NO donor that mimics endogenous NO production by NO synthase 2, in a dose-dependent manner (Fig 2D). Finally, we checked that cellular iron chelation with SIH induced stabilization of HIF-1α and expression of its target CAIX (Fig 2D), as previously observed by others with DFO treatments or with an NO donor [33,34].

Next, we further investigated whether VDAC_25K_ corresponds to the previously identified hypoxia-induced VDAC1-ΔC [17] by using a VDAC antibody (VDAC1-Nter) that specifically recognizes an epitope within the N-terminal region of human VDAC1 (Fig 2E). Using this specific antibody, we still detected the accumulation of VDAC_25K_ when ISCU was depleted in HeLa cells grown in normoxia without CAIX induction (Fig 2E *lane 7*). We unambiguously showed that this shorter form of VDAC is VDAC1-ΔC, which accumulated also under conditions that activate HIF-1α (presence of CAIX), such as 1% hypoxia (*lane 2*), SIH (*lane 4*) or NO (*lane 5*) treatments. We observed that knock-down of ISCU further increased VDAC1-ΔC accumulation when cells were grown under hypoxic conditions (Fig 2F, compare *lanes 3* and *4*) at a level similar to that observed when cells were treated with DFO (Fig 2F *lane 5*). Thus, for the first time, by depletion of the mitochondrial iron importer MFRN2 or of proteins of the ISC machinery, we were able to induce in normoxia a C-terminal truncation of VDAC1 similar to what was observed in cancer cells grown in hypoxia. Thus, for greater clarity, we will use the term VDAC1-ΔC previously employed, for the VDAC_25K_ form we observed in our experiments.

### Components of the core ISC assembly machinery are downregulated in hypoxia

**Fig 3:**
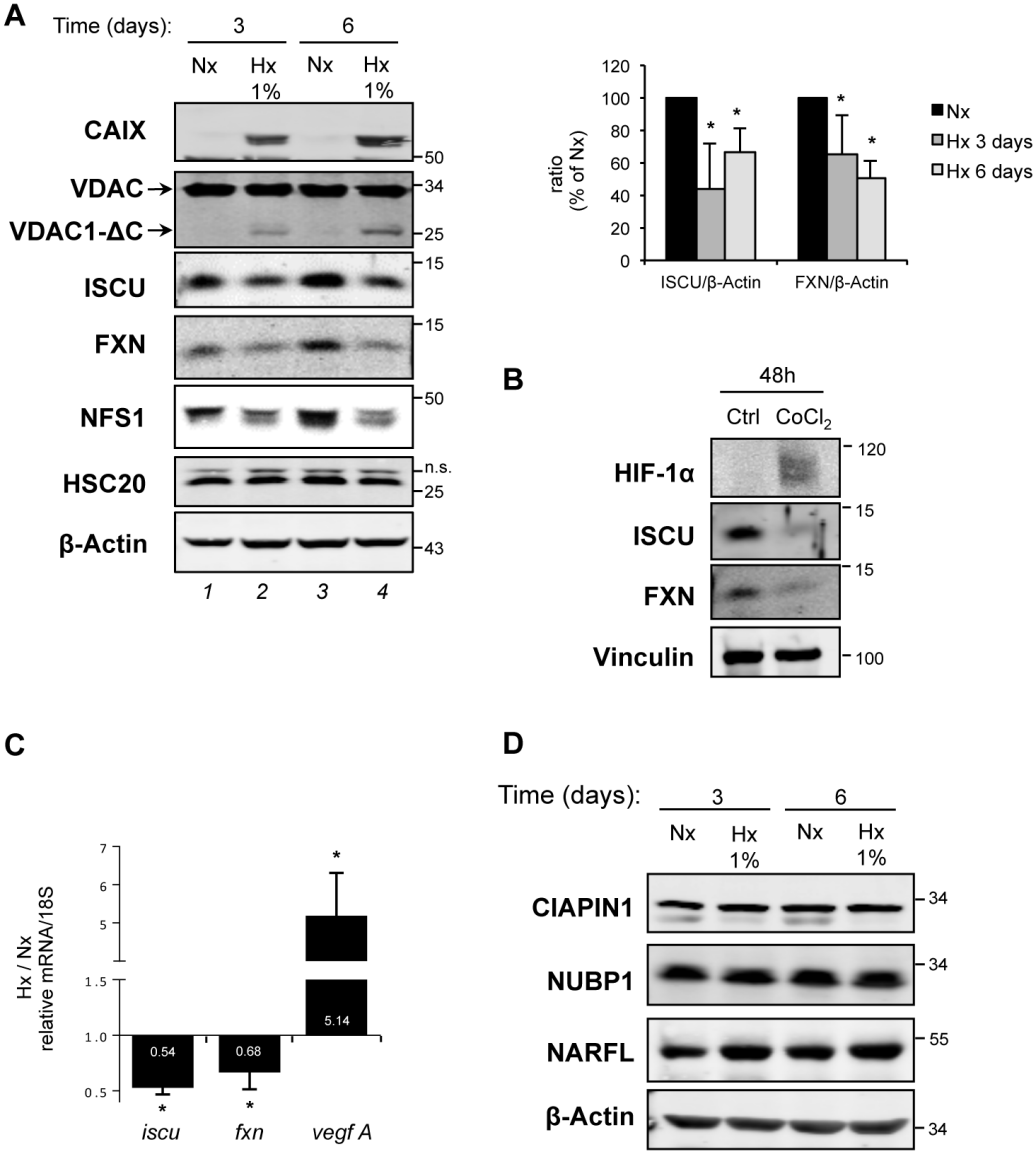
Impact of hypoxia on proteins of the mitochondrial ISC assembly machinery. (A) Total protein extracts from HeLa cells grown in normoxia (Nx, 21% O_2_) or hypoxia (Hx, 1% O_2_) conditions for the indicated times were analyzed by immunoblotting using VDACs poly antibody and anti-CAIX, -ISCU, -FXN, -NFS1, -HSC20 antibodies. β-Actin was used as loading control. (*right panel)* Bar graph represents the amount of the indicated proteins relative to ß-actin level determined by quantification of n = 3 immunoblot analysis using the Odyssey System Imager. Mean and standard deviation of 3 independent experiments are shown (* p < 0.05, n = 3). (B) HeLa cells were either untreated or treated with CoCl_2_ for 2 days. Total proteins were analyzed by western blotting using anti-HIF-1α, -ISCU, -FXN antibodies. Vinculin was used as loading control. (C) HeLa cells grown in normoxia (Nx, 21% O_2_) or hypoxia (Hx, 1% O_2_) conditions for 3 days and mRNA levels of *iscu*, *fxn* and *vegf A* were determined by RT-qPCR and normalized to 18S ribosomal rRNA levels. The bar graph presents the ratio between the relative level of each mRNA under hypoxic and normoxic conditions with a logarithmic scale. Mean and standard deviation of 6 independent experiments are shown (* p < 0.05, n = 6). (D) Total protein extracts from HeLa cells grown under normoxic (Nx, 21% O_2_) or hypoxic (Hx, 1% O_2_) conditions for the indicated times were analyzed by immunoblotting using anti-CIAPIN1, -NUBP1, -NARFL antibodies. β-Actin was used as loading control.

It has previously been shown that hypoxic exposure of cells leads to downregulation of the scaffold ISCU at a post-transcriptional level by miR-210, one of the most hypoxia-sensitive miRNAs, in human pulmonary arterial endothelial cells (HPAEC) [25,35]. Here, we showed that ISCU was also diminished at the protein level in another human cell type, the cervical cancer cell line HeLa, grown under hypoxic conditions for 3 to 6 days (Fig 3A *lanes 2* and *4*). Our results and those already mentioned above led us to investigate whether two other ISC components, frataxin (FXN) and NFS1, which form a complex with ISCU (the core ISC-assembly machinery), were affected by hypoxia. We observed that both proteins (FXN and NFS1) were downregulated in cells and that their low protein levels were maintained after 6 days in 1% O_2_ (Fig 3A). In addition, comparable decrease in FXN and ISCU protein levels was confirmed by chemical stabilization of HIF-1α using the prolyl hydroxylase inhibitor cobalt chloride (CoCl_2_) (Fig 3B). By RT-qPCR analysis, we also showed a marked decrease of not only ISCU but also FXN at the mRNA level in hypoxia (Fig 3C). In parallel, HIF-1α activation was confirmed by the increase of the mRNA level of its target gene *vegf A* (Fig 3C). Finally, we decided to investigate whether additional proteins involved in Fe-S cluster biogenesis, which are part of either the late step of mitochondrial ISC assembly (HSC20) or of the cytosolic Fe-S assembly CIA machinery (CIAPIN1, NUBP1 and NARFL), were also regulated in hypoxia. In contrast to what we observed for the upstream mitochondrial ISC components (NFS1, ISCU and FXN), protein levels of HSC20, CIAPIN1, NUBP1 and NARFL were unchanged in cells cultivated under hypoxic conditions as compared to those cultivated under normoxic conditions, even after 6 days (Fig 3A and 3D). In conclusion, we showed that ISCU, FXN and NFS1, which belong to the core ISC complex, were specifically downregulated in hypoxia in HeLa cells.

### Deficiency of mitochondrial Fe-S cluster assembly prevents caspase-3 activation

**Fig 4:**
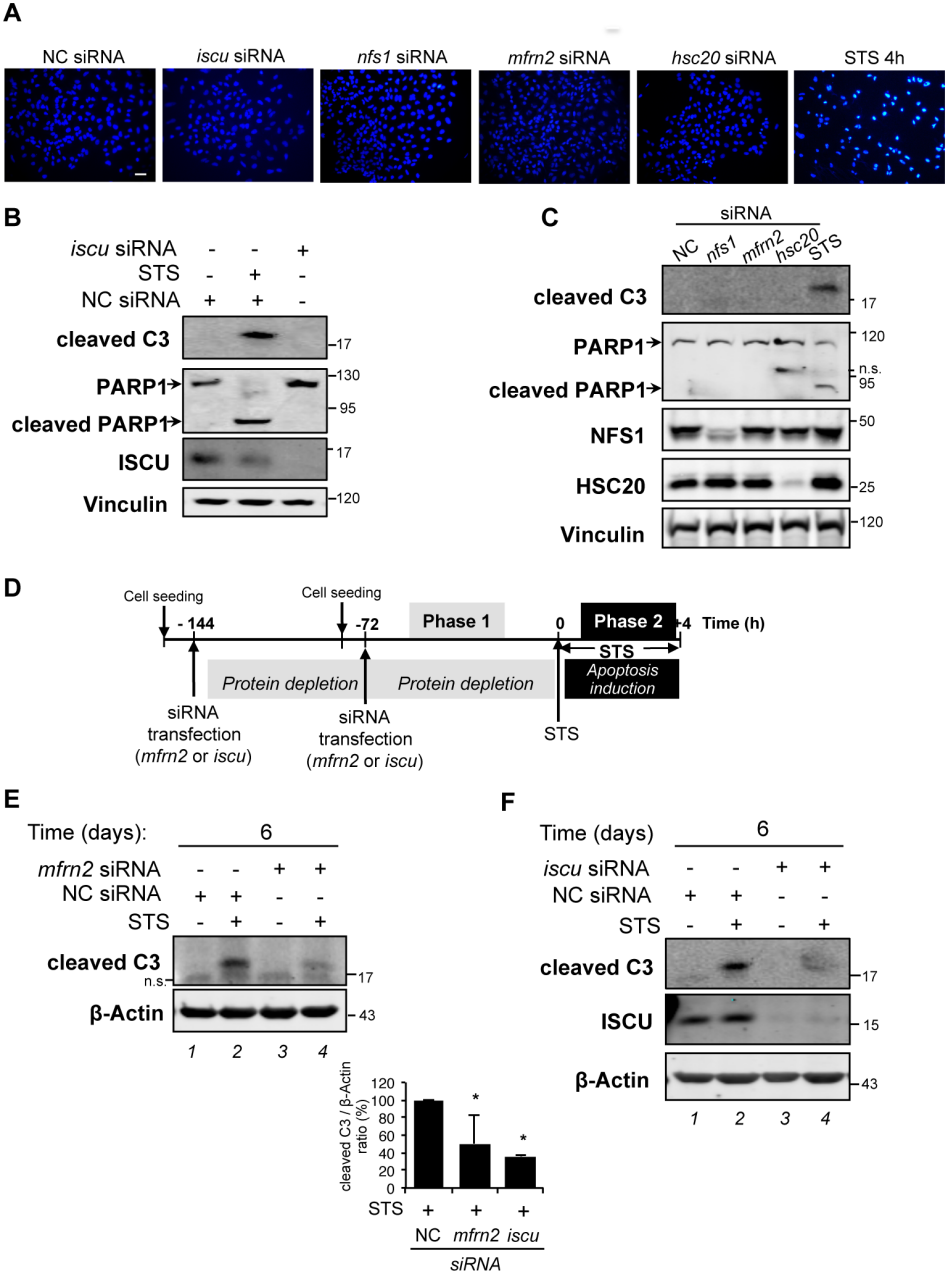
Protein depletion in the mitochondrial ISC machinery confers cell protection against STS-induced apoptosis. (A) HeLa cells were transfected with negative control (NC) or *iscu-*, *nfs1-*, *mfrn2-* or *hsc20*-siRNA for 6 days. Cells treated with staurosporine (STS) for 4 h were used as positive control for apoptosis induction. After staining with Hoechst 33342, cells were analyzed by epifluorescence microscopy. Scale bar: 100 μm. (B) HeLa cells were either transfected with NC or *iscu*-siRNA for 3 days or treated with STS for 4 h. Total protein extracts were analyzed by immunoblotting using antibodies against cleaved caspase 3 (C3), PARP1 and ISCU. Vinculin was used as loading control. (C) Cells were transfected with negative control (NC) or, *nfs1-*, *mfrn2-* or *hsc20*-siRNA for 6 days or STS-treated for 4 h. Total protein extracts were analyzed by immunoblotting using antibodies against cleaved caspase-3, PARP1, NFS1 and HSC20. Vinculin was used as loading control. (D) Schematic representation of the protocol used. *Phase 1*—HeLa cells were seeded the day before the transfection with *mfrn2-*, *iscu-* or NC siRNA and incubated for 6 days (144 hours) under normoxic conditions (21% O_2_) with a transfection every 3 days. *Phase 2* –Cells were either untreated or treated with STS for 4 h. (E and F) Total protein extracts were analyzed by immunoblotting using antibodies against cleaved caspase-3 (C3) and ISCU. β-Actin was used as loading control. (*Lower panel)* The bar graph presents the ratio between the amounts of cleaved C3 over ß-actin determined by quantification of the immunoblot. Mean and standard deviation of n = 3 (*iscu*-siRNA) and n = 4 (*mfrn2*-siRNA) independent experiments (* p < 0.05).

In this study, we have shown the accumulation of truncated VDAC1-ΔC when either the mitochondrial iron importer MFRN2 or components of the mitochondrial ISC assembly machinery were depleted in living cells. Considering previous reports showing that, in hypoxia, accumulation of VDAC1-ΔC is linked to cell chemoresistance [17], we asked whether the depletion of MFRN2 or of proteins of the mitochondrial ISC assembly machinery could also intrinsically modify cellular sensitivity to drug-induced apoptosis. To solve this issue, we first checked whether MFRN2 deficiency and protein depletion in the ISC machinery induce apoptosis *per se* by analyzing nuclear morphology and two apoptotic events, namely the cleavages of caspase 3 and of poly (ADP-ribose) polymerase-1 (PARP1). No apoptosis was observed by epifluorescence microscopy when nuclei were stained with Hoechst 33342 after 6 days of *iscu*, *nfs1*, *mfrn2* or *hsc20* silencing by siRNA (Fig 4A). In contrast, a 4 h-treatment with staurosporine (STS 4 h)–a classic apoptotic drug used here as apoptotic inducer control–led to enhanced fluorescence intensity, indicating nucleus fragmentation and condensation (Fig 4A). Using immunoblotting analysis, we then showed cleavage, and consequently activation, of the apoptotic effector caspase 3 (cleaved C3) by STS treatment, but not by the knock-down of ISCU (Fig 4B). Apoptotic caspase 3 activation leads to the proteolytic cleavage of PARP1 into a 90 kDa fragment [36,37]. We showed that PARP1 cleavage was detected only after STS treatment, but not in cells with a reduced level of ISCU (Fig 4B). Similar results concerning caspase 3 and PARP1 were observed with cells depleted for MFRN2 or the two other members of the ISC machinery, NFS1 and HSC20 (Fig 4C).

We then determined whether depletion of proteins involved in the maturation of Fe-S clusters conferred cell resistance to induction of apoptosis. For this purpose, we chose to perform knock-down of MFRN2 and of ISCU by siRNA for 6 days, which induced the highest level of accumulation of VDAC1-ΔC in our conditions (Fig 1B and S4A and S4B Fig). Once cells were ISCU- or MFRN2-depleted, we treated them with STS for 4 additional hours (Fig 4D). In these conditions, the depletion of MFRN2 (Fig 4E) or ISCU (Fig 4F) decreased caspase 3 activation two-fold compared to cells transfected with a negative control siRNA (NC siRNA) (Fig 4E and 4F *lanes 2* and *4*, and lower bar-graph). Our data showed that depletion of MFRN2 and of proteins of the mitochondrial ISC machinery confers cell resistance to effector caspase activation by an apoptotic treatment like STS, as demonstrated previously with cancer cells grown under hypoxic conditions [20].

### CISD2 depletion leads to the accumulation of VDAC1-ΔC in normoxia

We next attempted to identify the link between ISC machinery downregulation and accumulation of the anti-apoptotic VDAC1-ΔC. First, we asked whether VDAC1 could harbor a still unidentified Fe-S cluster that could be necessary to protect it against truncation, because of the presence in VDAC1, 2 and 3 of residues, including cysteine residues, which could coordinate such a cofactor. The aerobic lability of Fe-S clusters makes their identification difficult. Then, the anaerobic *in vitro* reconstitution of Fe-S clusters is a ‘gold standard’ method to assess the ability of a protein to bind to such clusters [38,39]. Thus, in order to determine if VDAC could accommodate an Fe-S cluster, we first overexpressed human VDAC1 and VDAC2 in *E*. *coli* and purified them on lauryldimethylamine oxide (LDAO) detergent micelles using already published protocols [40–42]. We then performed chemical Fe-S cluster reconstitution using the purified proteins by addition of ferric salt, sulfide under reducing and anaerobic conditions using classic protocols [38,43] and observed that purified VDAC1 and VDAC2 were unable to insert an Fe-S cluster at least in these conditions.

**Fig 5:**
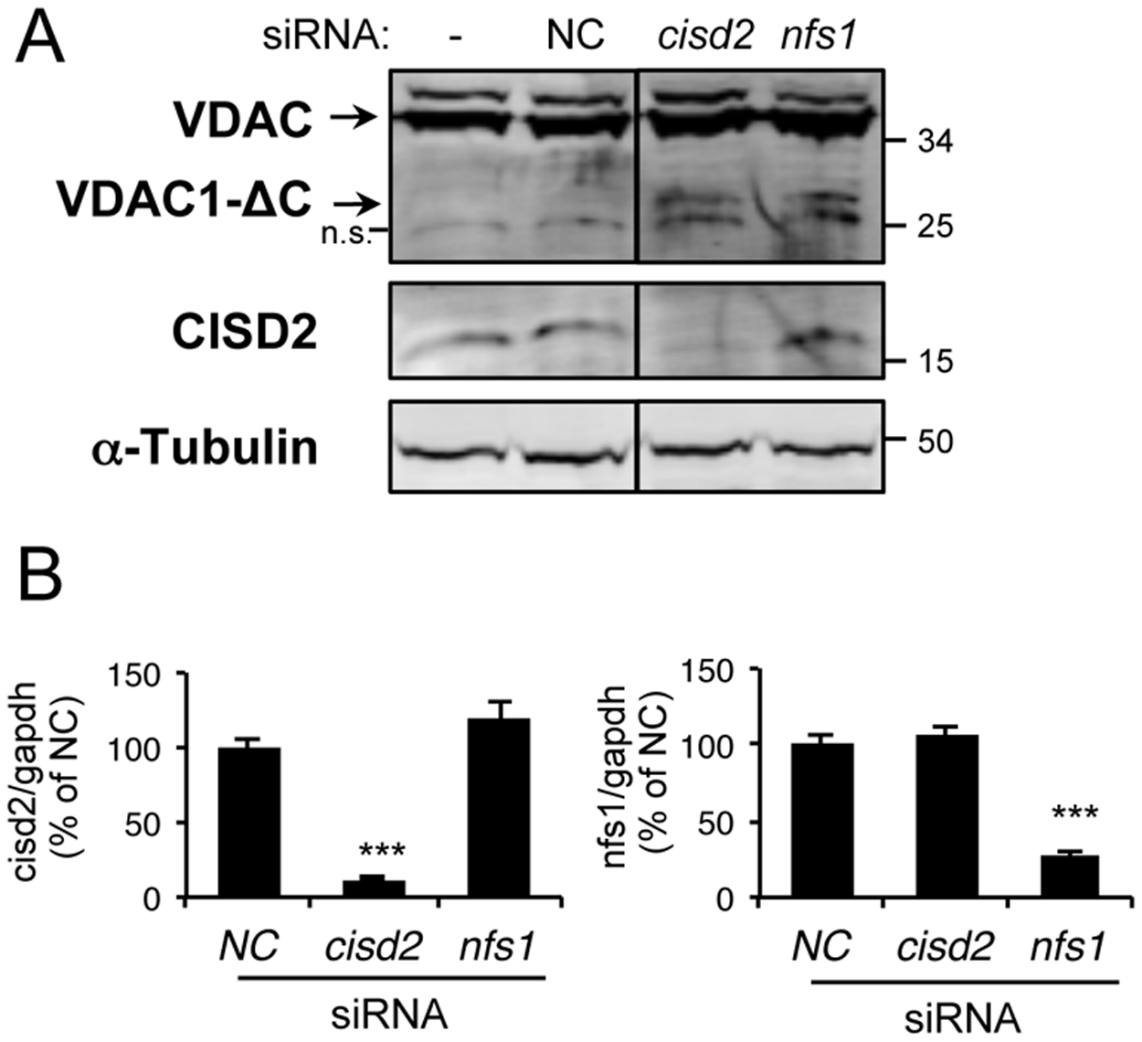
Knock-down of CISD2 induces the accumulation of the truncated VDAC1 form. (A) Total protein extracts of either non-transfected (-) or transfected HeLa cells for 3 days with scramble (NC)-, *cisd2*-, or *nfs1*-siRNA were analyzed by immunoblotting using antibodies against VDACs poly, CISD2, and α-tubulin, which was used as loading control. Eight independent experiments were performed and a representative western blot is shown. (B) Total mRNA was extracted from HeLa cells transfected with scramble (NC)-, *cisd2*-, or *nfs1*-siRNA. Twenty-four hours after transfection, the mRNA levels of *cisd2* and *nfs1* were determined by quantitative RT-qPCR. Data were normalized to *gapdh* mRNA levels. Means ± standard deviation of n = 4 independent experiments are presented (*** p < 0.001).

Our data led us to look at known Fe-S proteins in which impaired maturation could be linked to the cleavage of VDAC1. CISD2 is a member of the CDGSH iron-sulfur domain (CISD) family that has recently been implicated in the regulation of autophagy and apoptosis through its interaction with BCL-2 and beclin1 at the endoplasmic reticulum (ER) [22]. At the ER, CISD2 interacts with the inositol 1,4,5-triphosphate receptor (IP_3_R), which interacts with HSPA9 (or GRP75/mortalin), the chaperone protein also involved in the mitochondrial ISC machinery. Strikingly, HSPA9 directly interacts with VDAC1 at the OMM [21], suggesting a potential link between the ER Fe-S protein CISD2 and mitochondrial VDAC1. Interestingly, although located at the ER membrane, CISD2 knock-out in mice causes mitochondrial dysfunction [44,45] and elongation of mitochondria with extensive compact cristae remodeling [46,47]. Finally, the fibroblasts of a patient with a point mutation in *CISD2* present a more elongated mitochondrial network [48]. To determine if CISD2 is involved in VDAC1 truncation, we depleted HeLa cells for CISD2 using siRNA for 3 days. In parallel, we used either *nfs1* siRNAs as positive control for the VDAC1-ΔC accumulation, or scramble siRNA as negative control (NC). The depletion of CISD2 was confirmed both at the protein level by western blot and at the mRNA level by RT-qPCR (Fig 5A and 5B). Here, we observed that knock-down of CISD2 leads to VDAC1-ΔC accumulation at a level similar to that observed in NFS1-depleted cells. Our findings led us to propose that CISD2 is an important link between deficiency in mitochondrial Fe-S cluster protein biogenesis and C-terminal truncation of VDAC1 in normoxia.

## Discussion

**Fig 6:**
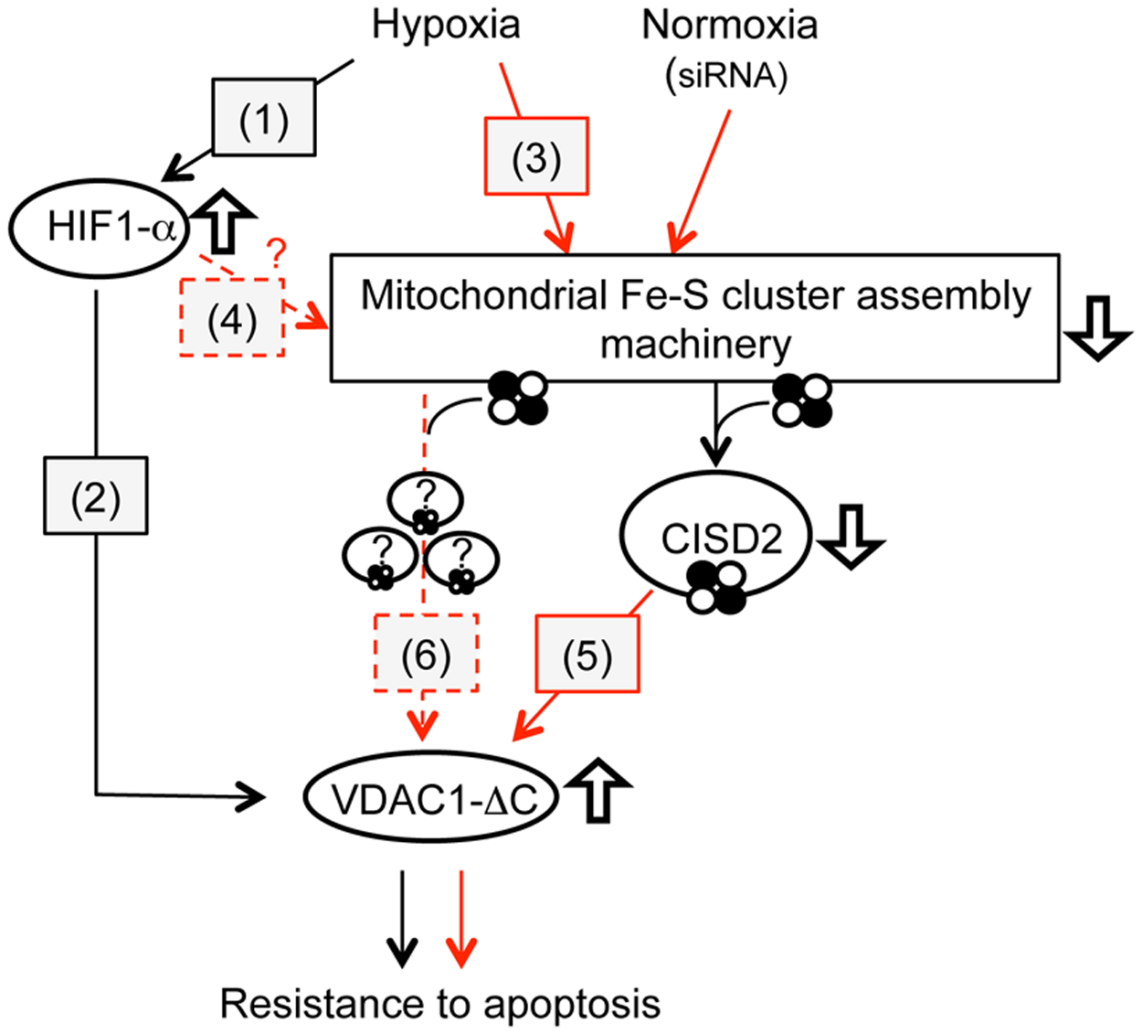
Downregulation of mitochondrial Fe-S cluster assembly leads to C-terminal VDAC1 truncation and subsequent resistance to pro-apoptotic treatments. Red and black arrows represent links unveiled in the present study and in previous studies [17,18], respectively. Hypoxia stabilizes and activates HIF-1α (1) and leads to drastic changes in mitochondrial morphology [17] and VDAC1-ΔC accumulation [18](2). In the present study, we showed that hypoxia leads to the downregulation of NFS1, FXN and ISCU, three components of the mitochondrial ISC core machinery (3). Whether this regulation directly involves HIF-1α remains to be explored (4). Subsequently, deficit in Fe-S cluster biogenesis leads to the loss of the mitochondrial network, appearance of enlarged mitochondria and accumulation of VDAC1-ΔC. We identified the MAM-anchored Fe-S protein CISD2 as a key protein for the cellular fate of VDAC1 (5). We still cannot exclude that CISD2 is the only Fe-S protein involved in this process (6). Interestingly, when components of the ISC core machinery were silenced by siRNA in hypoxia, VDAC1-ΔC levels were increased compared to hypoxia alone.

When cells are grown under limiting oxygen conditions (hypoxia), the transcriptional activator HIF-1α (hypoxia-inducible factor-1) accumulates, is transported to the nucleus and upregulates expression of hypoxia target genes. Moreover, under these growth conditions, the morphology of mitochondria changes from a tubular network to an unusual enlarged morphology (giant mitochondria) due to imbalance between mitochondrial fusion and fission. These mitochondrial modifications are associated with resistance to cell death [20], accumulation of a C-terminal truncated form of VDAC1 (VDAC1-ΔC) and cell resistance to chemotherapy-induced apoptosis [17–19]. Moreover, VDAC1-ΔC has been detected in tumor tissues of patients with early (stage I) and late (stage III) stage tumors. Its accumulation has thus been considered as a marker of tumor progression [17] and has, up to now, been associated with the accumulation of HIF-1α transcription factor due to hypoxic conditions. In addition to oxygen-dependent stabilization, HIF-1α (stability and activity) is also regulated by diverse mechanisms including ERK phosphorylation and nitric oxide [33]. Recently, it was shown that deficiency in ERK-dependent phosphorylation induces the relocation of hypoxia-induced HIF-1α to the OMM, its interaction with HSPA9 and C-terminal truncation of VDAC1 [49]. Here, for the first time, by impairing Fe-S cluster biogenesis by depletion of proteins involved in mitochondrial iron import (MFRN2) or in the mitochondrial ISC machinery (ISCU, NFS1 and HSC20), we demonstrate the accumulation of VDAC1-ΔC under normoxic conditions (21% O_2_) without HIF-1α/HIF-2α stabilization/activation in HeLa cells (Fig 6). After a 3-day siRNA extinction, the cleavage of VDAC is already clearly visible by immunoblotting in the case of *iscu* siRNA (apparition of a faint band at 25 kDa in Fig 2E) but the cleavage is stronger after 6 days of extinction (Fig 1B and S4 Fig). Depletion of ISC protein induces roughly 5% of truncated VDAC1 as we obtained under hypoxic conditions. VDAC1 is an abundant mitochondrial protein and this relatively small proportion of VDAC1 truncation is sufficient to modify drastically the mitochondrial phenotype (formation of giant mitochondria) and to induce resistance to pro-apoptotic drug. Similar giant mitochondria were observed when ISCU was depleted in the breast cancer cell line MDA-MB-231. Previous studies have shown that downregulation of ISC machinery can occur in physiological conditions. Indeed, in IFN-gamma- and LPS-activated macrophages the mitochondrial ISC machinery is downregulated [50] and the oxidative stress level is high. It is therefore tempting to speculate that inflammatory macrophages could accumulate VDAC1-ΔC, which might prevent the undesired activation of apoptosis in macrophages.

### Hypoxia downregulates several proteins of the mitochondrial ISC machinery

In solid tumors, cells grow rapidly and cause the formation of regions of hypoxia, which induces a cellular adaptive response leading to a metabolic shift involving the stabilization of HIF-1α, one of the major signatures of this cellular response. One consequence of HIF-1α activation is the upregulation of miR-210 [51], which is associated with cancer progression and poor prognosis [29]. It was previously shown that expression of miR-210 in hypoxia downregulates the ISC platform ISCU [35,52], promotes Fe-S cluster deficiency [25], and suppresses iron homeostasis-related proteins [53]. Here, we demonstrate that hypoxia affects not only ISCU, but also at least two other proteins of the mitochondrial ISC core machinery, namely FXN and NFS1. This downregulation of the ISC core machinery in hypoxia could explain the crosstalk between HIF-1α activation and ISC deficiency in VDAC1-ΔC formation and, consequently, the higher VDAC1 cleavage in hypoxia and dysregulation of Fe homeostasis/Fe-S cluster maturation (Fig 6).

### VDAC does not seem to harbor an Fe-S cluster

We decided to investigate the molecular mechanisms linking Fe-S cluster maturation and VDAC truncation, and to identify which Fe-S, affected by mitochondrial ISC deficit, induces VDAC truncation. First, we hypothesized that VDAC might be an Fe-S protein and that the assembly of its Fe-S cluster could protect it from truncation. Fe-S clusters are often necessary to maintain protein structure [54]. Contrarily, in glutamine phosphoribosylpyrophosphate aminotransferase (GPAT), the insertion of its cluster is necessary for the cleavage of the N-terminal residues to generate its mature form [55]. Fe-S clusters are typically bound to the polypeptide through side chains of cysteine residues, but non-cysteinyl coordination involving mostly histidine residues is also found [56]. Analysis of human VDAC sequences shows that VDAC1 harbors 2 cysteines while 9 and 6 are found in VDAC2 and VDAC3, respectively. In the VDAC3 isoform, cysteines form disulfide bonds involved in the regulation of pore size in response to redox variations [57–59]. Cysteines present in VDAC2 are clearly important for the overall architecture of the protein [60,61]. In VDAC1, cysteine residues are not essential for the channel activity of the protein, but could be important for its oligomerization [62]. In addition to the cysteine residues, VDAC1 and VDAC2 also possess 3 and 4 histidine residues, respectively. We purified human VDAC1 and VDAC2 on micelles under aerobic conditions and tried chemically to reconstitute an Fe-S cluster in both proteins under anaerobic and reducing conditions. In both cases, a cluster could not be introduced into purified proteins. Thus, we have no indication that VDAC could harbor an Fe-S cluster.

### Fe-S protein CISD2, a key player in VDAC1 truncation

We focused on Fe-S proteins in the close environment of VDAC1 and identified CISD2 as a promising candidate. CISD2 belongs to the CDGSH iron-sulfur domain (CISD) family and is anchored to the ER at the level of contact sites with the mitochondria, within MAM (mitochondria-associated membrane) microdomains, and with its Fe-S cluster in the cytosol. Like other Fe-S proteins, maturation of the CISD2 cluster depends on, at least, the mitochondrial ISC machinery. Thus, a flaw in the mitochondrial ISC affects the maturation of its cluster (Fig 6). On the one hand, CISD2 interacts with BCL-2 at the ER and affects its interaction with the tumor suppressor beclin1. Thus, CISD2 is a regulator of the initiation of autophagy in conditions of nutrient deprivation [22]. On the other hand, this protein interacts with IP_3_R [22], which forms a protein complex with HSPA9 and VDAC1 [21]. In addition, mutation [48] or knock-out [46,47] of *cisd2* induced elongation of mitochondria. We found out that CISD2 depletion in HeLa cells induced VDAC truncation to an extent similar to ISC machinery depletion. Thus, we propose that CISD2 could be a link between deficiency in Fe-S cluster protein biogenesis and cleavage of VDAC1 in normoxia (Fig 6). Moreover, this study confirms the critical role of CISD2 at the crossroads between the apoptosis and autophagy regulation pathways [63]. We know that its Fe-S cluster is redox active and that at least some of its biochemical properties are regulated by the redox state of its cluster [64]. More biochemical studies are needed to describe finely how CISD2 can regulate the autophagy/apoptosis pathways and the exact role of its cluster in this regulation.

### Use of iron chelator in cancer treatment?

Finally, we would like to address the clinical use of iron chelators such as desferrioxamine (DFO, Desferal^Ѓ^), deferiprone (Ferriprox^Ѓ^) and deferasirox (Exjade^Ѓ^) for patients with iron overload disorders [65]. Currently, some of these agents (such as DFO) are also under preclinical investigation as anticancer chemotherapeutics due their ability to arrest tumor growth [66,67,68] and to lower iron in the body [69]. Increased tumor resistance to classic chemotherapeutic agents is observed when iron levels rise [65]. Moreover, high iron availability is necessary for cancer cells to maintain their intensive proliferation [65]. However, in this work, we demonstrated that treatments of HeLa cells with at least two iron chelators induce rapid and intense accumulation of anti-apoptotic VDAC1-ΔC. In conclusion, these results raise the question of the risk associated with chemotherapy resistance by the induction of VDAC1 cleavage, due to combined chemotherapeutic treatments with iron chelators.

## Supporting information

S1 Fig *iscu* siRNA treatment did not affect ΔΨm. HeLa cells were transfected for 6 days with either NC (left) or *iscu* (right) siRNA. Mitochondrial membrane potential was studied using JC-1 dye and flow cytometry analysis. JC-1 probe selectively enters mitochondria and changes color from red to green as a sign for ΔΨm decreases. Representative results from 3 independent studies are presented.
(PDF)

S2 Fig Depletion of IscU in the breast cancer cell line MDA-MB-231 leads to the formation of giant mitochondria. MDA-MB-231 cells were transfected with either negative control (NC, Neg. contrl) or *iscu* siRNA for 6 days. (A) Epifluorescence microscopy after DAPI and CMXRos stainings to visualize nuclei and mitochondria, respectively. Scale bar: 10 μm. (B) The mRNA level of *iscu* was determined by RT-qPCR 24 h after transfection. Data are normalized to 36B4 mRNA levels and represented as a percentage of NC±S.D.
(PDF)

S3 Fig Cells grown under hypoxic conditions present enlarged mitochondria. Confocal microscopy of HeLa cells grown under normoxic (Nx, 21% O_2_) or hypoxic (Hx, 1% O_2_) conditions for 5 days on glass coverslips. Cells were treated with CMXRos probe before fixation. Scale bar: 10 μm. Lower panels show higher magnification of the part of the upper panel image is delineated by a white square.
(PDF)

S4 Fig Time course studies of protein depletion in the mitochondrial ISC assembly machinery leading to the accumulation of VDAC1-ΔC. HeLa were either left untransfected or were transfected with *iscu-* (A), *mfrn2*- (B), *nfs1-* (C), *hsc20-* (D), or NC siRNA (A-D) for the indicated times (maintained for up to 9 days with two or three rounds of siRNA transfections). Total protein extracts were analyzed by immunoblotting using antibodies against VDACs and ISCU. β-Actin was used as loading control.
(PDF)

S5 Fig Level of *ca9* mRNA after mitochondrial ISC assembly depletion. HeLa cells were transfected with *nfs1-*, *iscu-*, *mfrn2*-, *hsc20-*, or scramble (NC) siRNA, and mRNA levels of *CA9* were determined by RT-qPCR, normalized to *36B4* mRNA levels and represented as fold increase ± S.D.
(PDF)

S6 Fig Depletions of protein of the ISC assembly machinery do not stabilize HIF-2α. (A) HIF-2α western blot analysis. HeLa cells were either transfected with *iscu-*, *mfrn2-*, *hsc20*-, or scramble (NC) siRNA for 6 days or treated with DFO for 16h. Total protein extracts were analyzed by immunoblotting using anti-HIF-2α, -ISCU, -HSC20 antibodies. Vinculin was used as loading control. (B) mRNA levels of *glut1* (black) and *oct4* (grey) mRNA, gene targets of HIF2α, were determined by RT-qPCR, normalized to *36B4* mRNA levels and represented as fold increase ± S.D compared to non-transfected. Non-transfected (NT), scramble (NC) and *iscu-*, *mfrn2-* or *hsc20-* siRNA transfected.
(PDF)

## Abbreviations

CIA: cytosolic Fe-S assembly
DETA-NO: diethylenetriamineNONOate
DFO: desferrioxamine
ER: endoplasmic reticulum
FAC: ferric ammonium citrate
FDX: ferredoxin
ISC: iron-sulfur cluster
MAM: mitochondria-associated membranes
NARFL: nuclear prelamin A recognition factor-like
NC: negative control
OMM: outer mitochondrial membrane
ROS: reactive oxygen species
SIH: salicylaldehyde isonicotinoylhydrazone
VDAC: voltage-dependent anion channel
