# Supplementary figures and images for: Dysfunction in the mitochondrial Fe-S assembly machinery leads to formation of the chemoresistant truncated VDAC1 isoform without HIF-1α activation
Source: PLoS One. 2018 Mar 29;13(3):e0194782. doi: 10.1371/journal.pone.0194782 (PMC5875801; doi:10.1371/journal.pone.0194782)

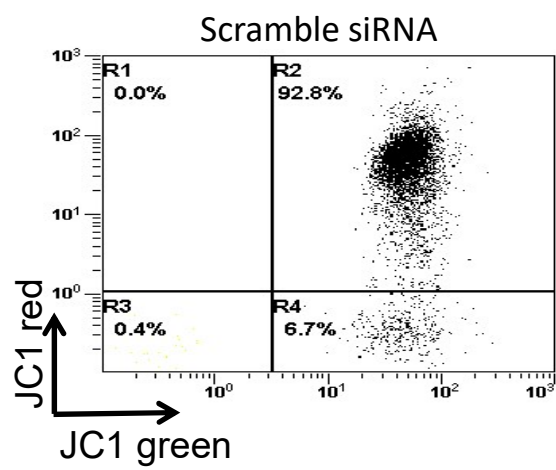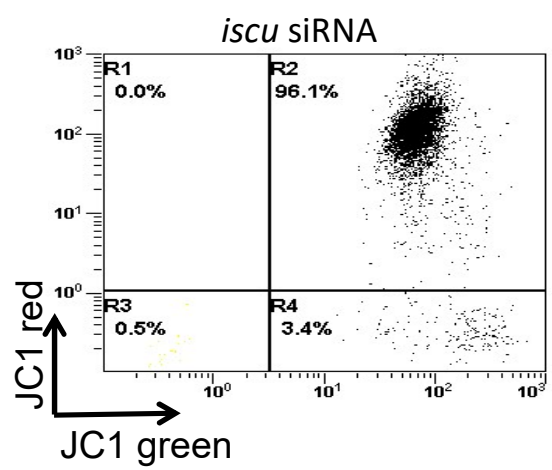

Figure S1.

Supplement: S1 Fig — HeLa cells were transfected for 6 days with either NC (left) or iscu (right) siRNA. Mitochondrial membrane potential was studied using JC-1 dye and flow cytometry analysis. JC-1 probe selectively enters mitochondria and changes color from red to green as a sign for ΔΨm decreases. Representative results from 3 independent studies are presented. (PDF) [file pone.0194782.s001.pdf]

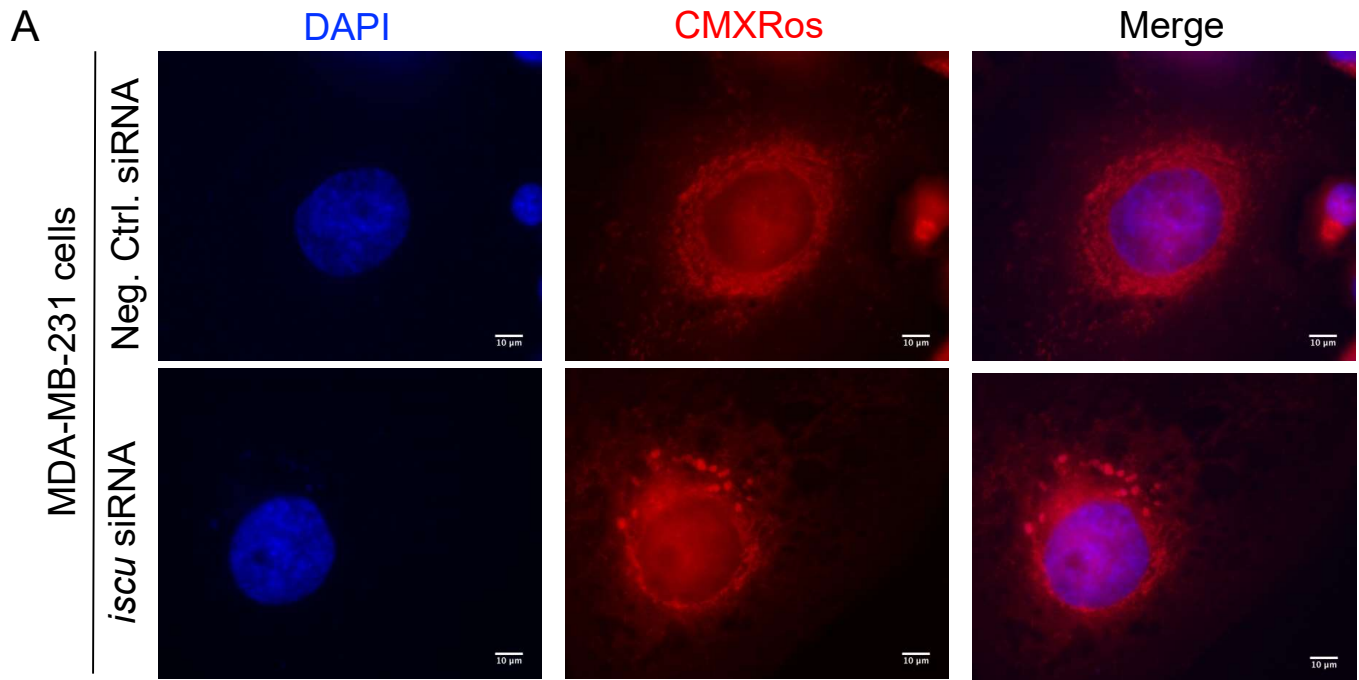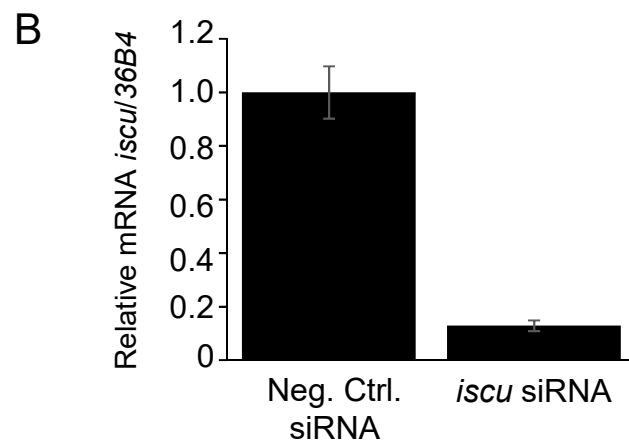

Figure S2.

Supplement: S2 Fig — MDA-MB-231 cells were transfected with either negative control (NC, Neg. contrl) or iscu siRNA for 6 days. (A) Epifluorescence microscopy after DAPI and CMXRos stainings to visualize nuclei and mitochondria, respectively. Scale bar: 10 μm. (B) The mRNA level of iscu was determined by RT-qPCR 24 h after transfection. Data are normalized to 36B4 mRNA levels and represented as a percentage of NC±S.D. (PDF) [file pone.0194782.s002.pdf]

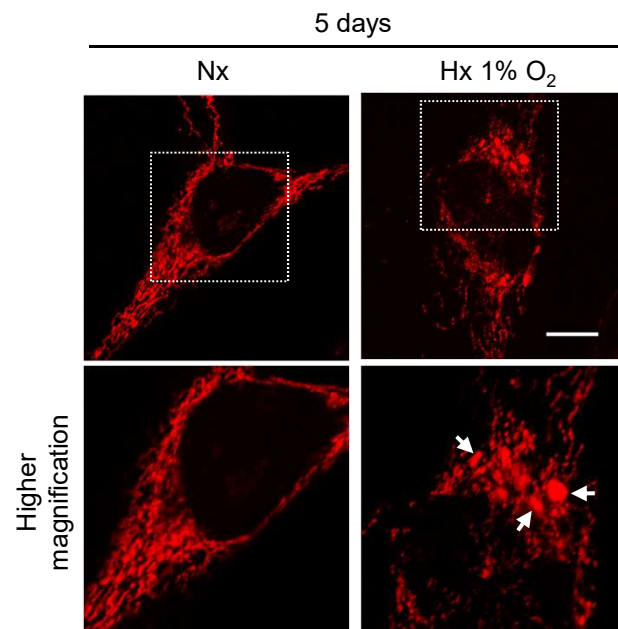

Figure S3.

Supplement: S3 Fig — Confocal microscopy of HeLa cells grown under normoxic (Nx, 21% O2) or hypoxic (Hx, 1% O2) conditions for 5 days on glass coverslips. Cells were treated with CMXRos probe before fixation. Scale bar: 10 μm. Lower panels show higher magnification of the part of the upper panel image is delineated by a white square. (PDF) [file pone.0194782.s003.pdf]

**A**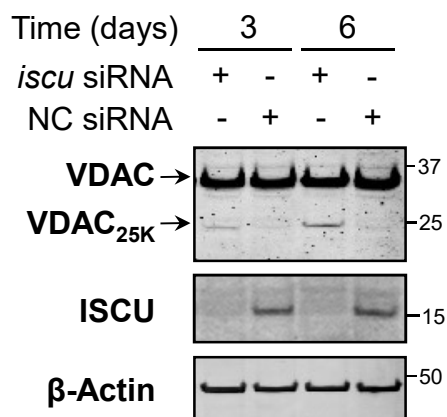**B**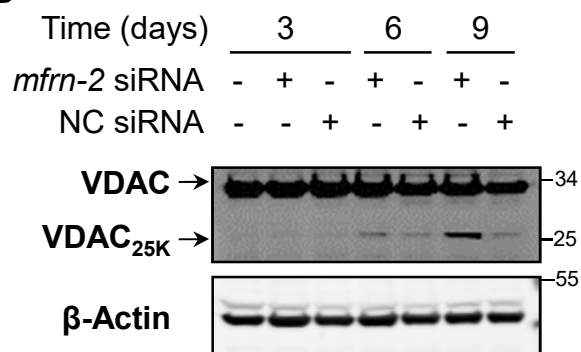**C**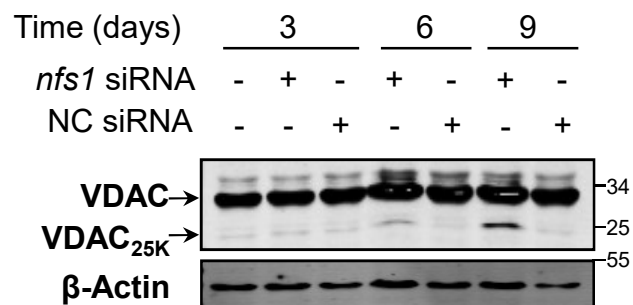**D**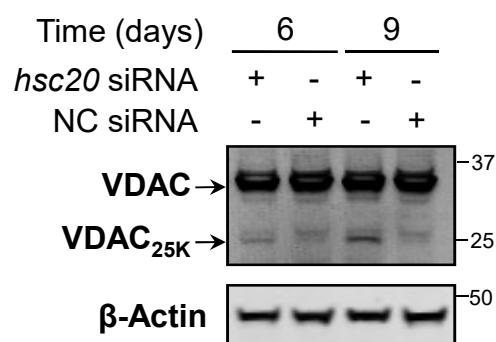

Figure S4.

Supplement: S4 Fig — HeLa were either left untransfected or were transfected with iscu- (A), mfrn2- (B), nfs1- (C), hsc20- (D), or NC siRNA (A-D) for the indicated times (maintained for up to 9 days with two or three rounds of siRNA transfections). Total protein extracts were analyzed by immunoblotting using antibodies against VDACs and ISCU. β-Actin was used as loading control. (PDF) [file pone.0194782.s004.pdf]

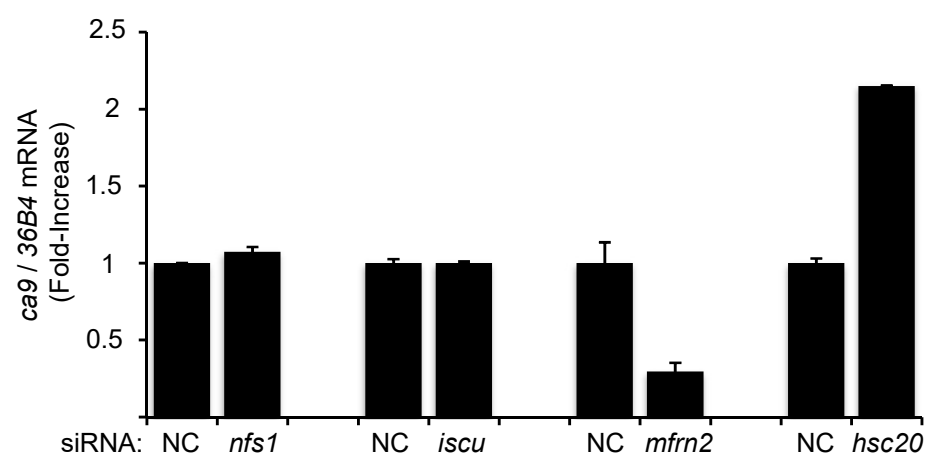

Figure S5.

Supplement: S5 Fig — HeLa cells were transfected with nfs1-, iscu-, mfrn2-, hsc20-, or scramble (NC) siRNA, and mRNA levels of CA9 were determined by RT-qPCR, normalized to 36B4 mRNA levels and represented as fold increase ± S.D. (PDF) [file pone.0194782.s005.pdf]

**A**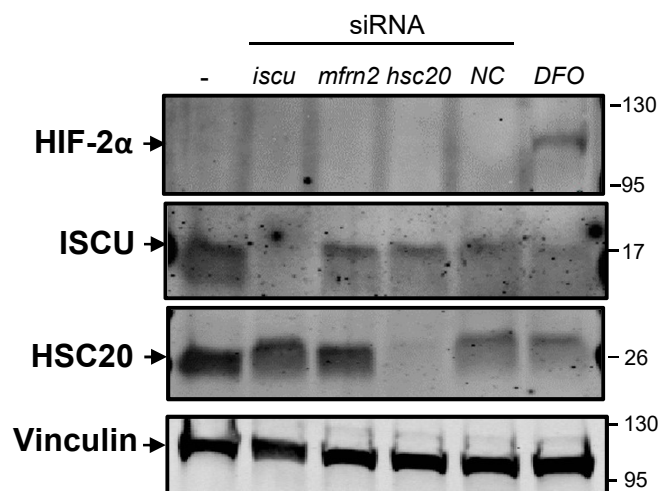**B**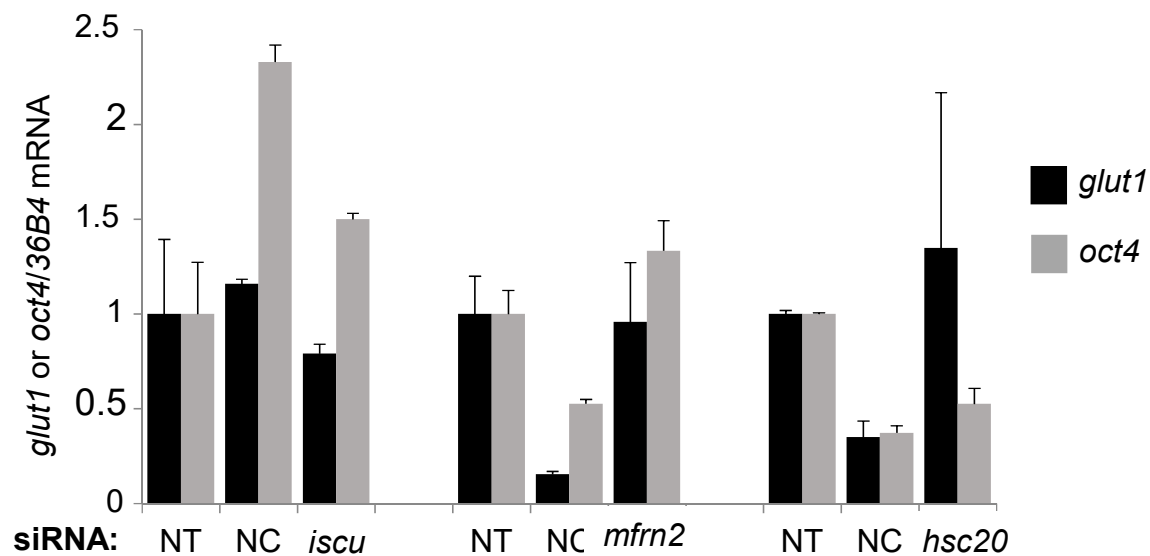

Figure S6.

Supplement: S6 Fig — (A) HIF-2α western blot analysis. HeLa cells were either transfected with iscu-, mfrn2-, hsc20-, or scramble (NC) siRNA for 6 days or treated with DFO for 16h. Total protein extracts were analyzed by immunoblotting using anti-HIF-2α, -ISCU, -HSC20 antibodies. Vinculin was used as loading control. (B) mRNA levels of glut1 (black) and oct4 (grey) mRNA, gene targets of HIF2α, were determined by RT-qPCR, normalized to 36B4 mRNA levels and represented as fold increase ± S.D compared to non-transfected. Non-transfected (NT), scramble (NC) and iscu-, mfrn2- or hsc20- siRNA transfected. (PDF) [file pone.0194782.s006.pdf]
